# Persistent inflammation worsens short-term outcomes in massive stroke patients

**DOI:** 10.1186/s12883-021-02097-9

**Published:** 2021-02-10

**Authors:** Duanlu Hou, Chunjie Wang, Xiaofei Ye, Ping Zhong, Danhong Wu

**Affiliations:** 1 Department of Neurology, Shanghai Fifth People’s Hospital, Fudan University, No. 801, Heqing Road, Shanghai, 200240 China; 2 Jiangchuan Community Health Service Center of Minhang District, Shanghai, China; 3 Department of Health Statistics, Second Military Medical University, Shanghai, China; 4 Department of Neurology, Shidong Hospital of Yangpu District, Shanghai, No.999, Shiguang Road, Shanghai, 200438 China

**Keywords:** Inflammation, Neutrophil-to-lymphocyte ratio, Stroke, Mortality, Functional outcome

## Abstract

**Background:**

Persistent inflammation is an important driver of disease progression and affects prognosis. Some indicators of inflammation predict short-term outcomes. The relationship between prognosis, especially mortality, and persistent inflammation in massive stroke has not been studied, and this has been the subject of our research.

**Methods:**

From April 1, 2017 to February 1, 2020, consecutive patients were prospectively enrolled. Clinical data, laboratory data, imaging data and follow-up infections morbidity were compared between 2 groups according to modified Rankin scale (mRS) scores (mRS < 3 and ≥ 3) at 1 month. The binomial logistic analysis was used to determine independent factors of 1-month prognosis. Short-term functional outcome, mortality and infection rates in massive stroke with and without persistent inflammation were compared.

**Results:**

One hundred thirty-nine patients with massive stroke were included from 800 patients. We found that admission blood glucose levels (*p* = 0.005), proportions of cerebral hemispheric (*p* = 0.001), posterior circulatory (*p* = 0.035), and lacunar (*p* = 0.022) ischemia were higher in poor outcome patients; neutrophil-to-lymphocyte ratio (odd ratio = 1.87, 95%CI 1.14–3.07, *p* = 0.013) and blood glucose concentrations (odd ratio = 1.34, 95%CI 1.01–1.79, *p* = 0.043) can independently predict the short-term prognosis in massive stroke patients. We also found that the incidence of pulmonary infection (*p* = 0.009), one-month mortality (*p* = 0.003) and adverse outcomes (*p* = 0.0005) were higher in patients with persistent inflammation.

**Conclusions:**

This study suggested that persistent inflammation is associated with poor prognosis, 1-month mortality and the occurrence of in-hospital pulmonary infection and that higher baseline inflammation level predicts short-term poor outcomes in massive stroke.

**Supplementary Information:**

The online version contains supplementary material available at 10.1186/s12883-021-02097-9.

## Background

Stroke, especially massive stroke (MaS), has a high incidence of mortality and morbidity [1]. Many factors, such as age, stroke severity, and National Institutes of Health Stroke Scale (NIHSS) scores at admission, have been proved to predict early stroke mortality [2]. Inflammation is involved in the development of ischemic stroke at all stages, from the early injury triggered by arterial occlusion to the late regenerative process underlying post-ischemic tissue repair [3]. Inflammation is initiated within a few hours and plays an important role not only in ischemic damage [4], but in cells such as endothelial progenitor cells in angiogenesis [5]. Local brain inflammation, resulting from neuronal damage in stroke, can aggravate a secondary injury and elicit and persist global brain inflammation [6]. The immune system can be activated by brain ischemia, and in turn promotes fatal infections especially pneumonia and urinary tract infection by an immunosuppressive effect that threatens the survival of 30% of patients after stroke [7]. The neutrophil-to-lymphocyte ratio (NLR), derived from the absolute neutrophil and absolute lymphocyte counts of a full blood count, can be a representative marker of systemic inflammation in conditions such as ischemic or hemorrhagic stroke [8], and various cancers [9]. Although a clinical scale predicting early death after stroke has been developed [2] and the usefulness of NLR in predicting poor functional outcomes has been proved in ischemic and hemorrhagic stroke [10, 11], whether systemic inflammation persisting a certain course of the disease predicts stroke mortality remains to be further explored, especially in MaS.

The present prospective study aimed to evaluate the impact of persistent, systemic inflammation in MaS on short-term outcomes such as mortality and nosocomial infections including pulmonary infection.

## Materials and methods

### Participants enrollment

Consecutive patients with either ischemic or hemorrhagic stroke were screened and selected from the Stroke Unit of Shanghai Fifth People’s Hospital (The Fifth People’s Hospital of Shanghai, Fudan university) between 1st April, 2017 and 1st February, 2020. Patients were included in the present study if they met the following criteria: (1) diagnosed with acute severe stroke (acute ischemic stroke (AIS) or intracerebral hemorrhage) within 24 h of onset; (2) aged 18 years or older; and (3) completed a head magnetic resonance imaging (MRI) or computed tomography (CT) within 24 h. AIS was diagnosed if there were new focal neurological deficits explained by relevant lesions detected on head diffusion-weighted imaging (DWI). Patients were excluded if they met the following criteria: (1) patients with previous AIS or present cerebral hemorrhage; (2) pregnant patients; (3) severe heart (with cardiac function in grade III or IV according to New York Heart Association or left ventricular ejection fraction < 40% in echocardiography), lung (with blood oxygen saturation less than 95% and symptoms of shortness of breath, cyanosis, and abnormal blood gas analysis), liver (serum alanine aminotransferase levels > 10-fold the upper limit of the reference range), kidney (serum creatinine > 443 μmol/L), and neoplastic diseases [4]; autoimmune diseases [5]; infection symptoms or signs were present at stroke onset.

According to the relevant guidelines, if the patients in the cohort met the criteria of thrombolysis, they would be treated by intravenous alteplase (the dosage = 0. 9 mg/kg × patient’s body weight (kg); 10% of the dosage was injected intravenously as a bolus within the first minute and the remaining 90% injected intravenously within 1 h) or neuro-intervention [12]. The inclusion/exclusion criteria of thrombolysis followed the Chinese guideline [12].

**Fig. 1:**
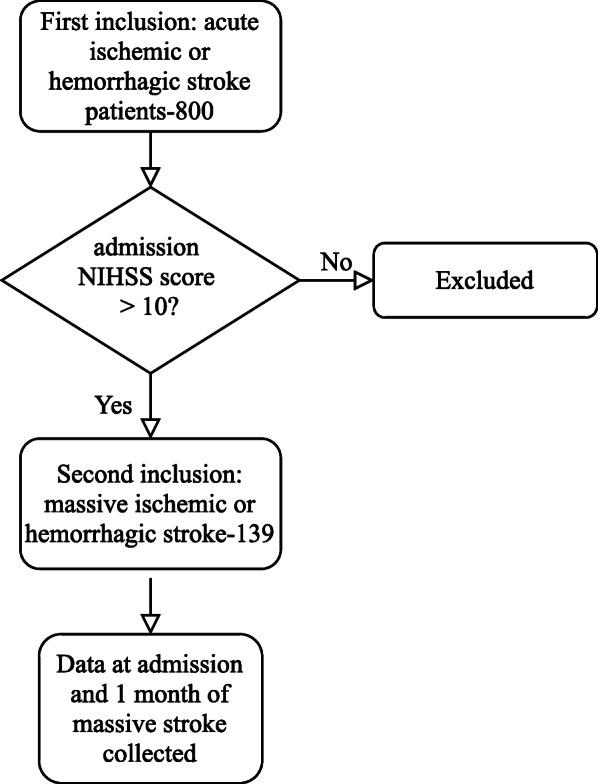
Flow chart of patients enrollment

Figure 1 showed the flow diagram of selecting patients. Written informed consent was obtained from all patients or their families. This study was approved by the Ethical Review Board of Shanghai Fifth People’s Hospital before the patient’s enrollment. Baseline data were collected from medical records including age, sex, and history of hypertension, diabetes mellitus (DM), atrial fibrillation (AF), dyslipidemia, NIHSS scores, systolic blood pressure (SBP), history of cigarette smoking, and alcohol drinking.

### Laboratory tests

All blood samples were collected via vacuum tubes, stored at 4 °C and tested by clinical laboratory technicians in hospital certificated laboratory within 2 h after collection. White blood count (WBC), neutrophil proportion (N), lymphocyte proportion (L), platelet count (P), serum total bilirubin, serum glucose, homocysteine (HCY), high-density lipoprotein (HDL), low-density lipoprotein (LDL), cystatin C (Cys C), sodium, potassium, D-dimer (DDI) and C reactive protein (CRP) concentrations were measured, and N, L, and DDI were measured at the time of admission and discharge. Neutrophil-lymphocyte ratio (NLR) was calculated as the ratio of neutrophil counts to lymphocyte counts (N/L). These indices were tested for AIS patients who would receive thrombolysis within 1 h after their presentation to the emergency department and before starting their treatments. For those who did not receive thrombolysis, blood samples were collected only once at the same time as the second blood sample collection for AIS patients who received thrombolysis, that was 6-7 am on the second day of admission.

### Clinical assessments

Each enrolled patient underwent a head 3-T MRI or 64-slice CT (Siemens, Forchheim, Germany) within 24 h after stroke onset. Severity was quantified by admission NIHSS scores and 1-month outcomes were quantified by 1-month mRS scores into 2 levels: favorable outcome (mRS < 3), and unfavorable outcome (mRS ≧ 3, = 6 means death) [13] and presentation of in-hospital infection (0 for no infection, 1 for infection). Massive stroke was defined as admission NIHSS score > 10. Definition of persistent inflammation is uncertain, we made the following definitions based on the relevant literatures [14–16] and the characteristics of our own subject population: The duration of persistent inflammation was chosen as more than 2 weeks in our study [15]. The NLR levels represent the inflammation severity. The baseline NLR and NLR at 14–28 days are measured, and it is determined that the patient is in a state of persistent inflammation when NLR levels at baseline and 30 days are > 5 [17]. The inclusion criteria for in-hospital infections are as follows: 1) no infection prior to hospitalization, 2) mainly includes lung and urinary tract infections, and 3) elevated routine blood parameters during hospitalization or a CT scan of the lungs suggestive of pneumonia with an elevated axillary temperature greater than 37.5 degrees Celsius. All included patients were followed up to get their mRS scores at 1 month and 3 months.

### Statistical analysis

SPSS 26.0 (IBM Corp., Armonk, NY) and GraphPad Prism 8 (GraphPad Software Inc., La Jolla, CA) were used to perform statistical analysis. Categorical variables were presented as frequency and percentage, and Pearson Chi-square test or Fisher’s exact test were used to compare categorical variables. For continuous variables, the Kolmogorov–Smirnov test was used to determine the data distribution. If the data demonstrates normal distribution, they are presented with mean ± SD and the Student t test is used for comparison. For data without normal distribution, median and interquartile range are shown and the Mann–Whitney U test is used for comparison. Significance level was set at α = 0.05 (two-tailed). Parameters showing a statistical trend (*p* < 0.1) or being proved a significant association in previous studies were included in a logistic model to identify parameters independently associated with functional outcome. The Hosmer–Lemeshow test was used to test the goodness of fit. Then, patients were dichotomized according to the identified cutoff value of NLR in a previous study [17], and 1-month mRS scores of different groups divided by cutoff value of NLR were tested by chi-square test or Mann–Whitney U test.

## Results

### Population characteristics

Over a period of 3 years, 139 patients with acute cerebral infarction or hemorrhage were included in the final analysis. These patients were divided into 2 groups according to modified Rankin scale scores, mRS < 3 means favorable outcome and mRS ≧ 3 unfavorable outcomes (Table 1). Participants with higher mRS scores have no difference with those with lower mRS scores in gender, age, smoking, and alcohol drinking. We found no difference in the prevalence of hypertension, diabetes mellitus, coronary heart disease, atrial fibrillation, liver disease and kidney disease between the 2 groups of patients. Also, in the comparison of blood pressure and blood glucose, we found that admission blood glucose levels were different between the 2 groups of patients, i.e., it was higher in the poor prognosis group (5.4 mmol/L, 4.6–6.3 vs 6.6 mmol/L, 5.3–8.2, *p* = 0.005); no difference was found in blood pressure. Among the laboratory indicators, inflammatory indicators were observed dynamically, and NLR was measured separately at admission and discharge, and it was found that among the many laboratory indicators, only the inflammatory indicators collected at 2-time points (*p* = 0.002; *p* = 0.028) were different in the 2 groups, other indicators such as sodium, potassium, total bilirubin, homocysteine and CRP were not statistically different. Imaging data analysis found that proportions of cerebral hemispheric (16.6% vs 49.3%, *p* = 0.001), posterior circulatory (33.3% vs 16%, *p* = 0.035), and lacunar (22.2% vs 6%, *p* = 0.022) ischemia but not cerebral bleeding were higher in poor outcomes patients. As for the comparison of treatments such as intravenous thrombolysis and arterial intervention, no difference in utilization rate between the 2 groups was found either. Finally, significant differences were found in hospital-related infections (lung infection, 31.7% vs 60.2%, *p* = 0.002; urinary tract infection, 0 vs 14.3%, *p* = 0.011) among the MaS patients between favorable outcomes and unfavorable outcomes group.

**Table 1:** Baseline characteristics of the study population and bivariate comparisons between favorable and unfavorable outcome patients

	Favorable functional outcome (*n* = 41)	Unfavorable functional outcome (*n* = 98)	*p* Value
Demographic data			
Male	23 (56.1)	49 (50)	0.512
Age ^a^	82 (13)	81 (11)	0.931
Smoker	9 (22)	17 (17.3)	0.526
Alcohol drinker	3 (7)	12 (12.2)	0.553
Medical history			
Arterial hypertension	34 (82.9)	72 (73.4)	0.232
Diabetes mellitus	10 (24.4)	39 (39.8)	0.083
Atrial fibrillation	9 (22.0)	29 (39.8)	0.357
Coronary artery disease	1 (2.4)	7 (7)	0.436
Liver disease	2 (4.9)	8 (8.2)	0.723
Kidney disease	1 (2.4)	7 (7.1)	0.436
Clinical characteristics			
Systolic BP, mmHg^b^	150 (132–162)	147 (138–164)	0.992
Blood glucose, mg/dl^b^	5.4 (4.6–6.3)	6.6 (5.3–8.2)	0.005^*^
Laboratory data			
Sodium, mmol/L^b^	141 (139–143)	140 (139–142)	0.084
Potassium, mmol/L^b^	4.0 (3.7–4.2)	3.8 (3.5–4.1)	0.144
CRP, mg/L	6 (3–10)	7 (3–11)	0.433
NLR at admission^b^	3.3 (2.3–4.9)	5.1 (4.0–8.3)	0.002^*^
NLR at discharge^b^	3.4 (2.0–5.2)	4.3 (2.7–6.3)	0.028^*^
Platelet, 10^9^/L^b^	202 (155–239)	193 (157–237)	0.900
LDL, mmol/L^b^	2.5 (2.1–3.3)	2.7 (1.9–3.4)	0.837
Total bilirubin, μmol/L^b^	12.1 (9.6–15.9)	15.2 (10.7–18.9)	0.080
Cystatin C, mg/L^b^	1.0 (1.1–1.2)	1.1 (1.0–1.3)	0.922
Homocysteine, μmol/L^b^	14.8 (11.5–19.3)	13.9 (11.1–19.9)	0.990
Imaging data			
Cerebral ischemia	36 (87.8)	81 (82.7)	0.448
Hemisphere	6 (16.6)	40 (49.3)	0.001^*^
Lobes	9 (25)	20 (24.6)	0.972
Brainstem/cerebellum	12 (33.3)	13 (16)	0.035^*^
Lacunes	8 (22.2)	5 (6)	0.022^*^
Cerebral hemorrhage	4 (9.8)	18 (18.4)	0.205
Basal ganglia	2 (50)	5 (28)	0.565
Lobes	1 (25)	12 (66)	0.264
Brainstem/cerebellum	1 (25)	1 (5)	0.338
Medication			
rtPA	4 (9.7)	11 (11.2)	0.799
Intervention	3 (7)	5 (5.1)	0.693
In-hospital infections			
Lung infection	13 (31.7)	59 (60.2)	0.002^*^
Urinary tract infection	0 (0)	14 (14.3)	0.011^*^

Abbreviations: *NLR* neutrophil-to-lymphocyte ratio; *BP* blood pressure; *LDL* low density lipoprotein; *CRP* C reactive protein; *rtPA* Recombinant Human Tissue Plasminogen A

Unless specified, values are numbers of patients (%)

^a^Mean (standard deviation)

^b^Median (interquartile range)

^*^Statistically significant

### Admission NLR can predict 1-month outcomes

Our multivariable regression analysis model (Table 2) suggests that NLR (odd ratio = 1.87, 95%CI 1.14–3.07, *p* = 0.013) and blood glucose concentrations (odd ratio = 1.34, 95%CI 1.01–1.79, *p* = 0.043) can independently predict the short-term prognosis of patients with the massive stroke, and in combination with Table 1, it can be concluded that the higher the baseline NLR level, the worse the 1-month functional outcomes of patients.

**Table 2:** Multivariable regression analysis for risk factors with favorable functional outcome

	Beta	SE	Wald	*p*	OR, 95% CI
Age	0.014	0.023	0.359	0.549	1.01, 0.97–1.06
Gender	−0.004	0.536	0.000	0.994	0.99, 0.35–2.85
NLR rank ^a^	0.555	0.264	4.438	0.035^*^	1.74, 1.01–2.92
SBP, mmol/L	0.003	0.015	0.042	0.837	1.00, 0.97–1.03
Glc, mmol/L	0.350	0.160	4.802	0.028^*^	1.42, 1.04–1.94
Bil, mmol/L	0.056	0.044	1.587	0.208	1.05, 0.97–1.15
UTI	1.389	1.222	1.293	0.256	4.01, 0.36–44.01
NEU	0.935	0.564	2.749	0.097	2.53, 0.84–7.69
Constant	−4.219	3.170	1.771	0.183	0.02, −

Abbreviations: *SE* standard error; *OR* odd ratio; *CI* confidence interval; *NLR* neutrophil-lymphocyte ratio; *SBP* systolic blood pressure; *Glc* blood glucose levels; *Bil* blood total bilirubin levels; *UTI* urinary tract infection; *NEU* pneumonia

^a^NLR at admission

*Statistically significant

### Persistent inflammation and 1-month outcomes

By comparing the incidence of patient outcomes including pulmonary infection, urinary tract infection, 1-month mortality and 1-month mRS in the group with and without persistent inflammation (Table 3), we found that the incidence of pulmonary infections (*p* = 0.009) as well as one-month mortality (*p* = 0.003) and adverse outcomes (*p* = 0.0005) was higher in patients with persistent inflammation than that without persistent inflammation.

**Table 3:** Comparisons of short-term outcomes between patients with and without persistent inflammation

Short-term outcomes	Persistent inflammation		*p* value	*RR*	*95%CI*
	With	Without			
Pulmonary infection	21 (77.8)	23 (46)	0.009^*^	1.7	1.2–2.4
Urinary tract infection	3 (11.1)	6 (12)	1	–	–
1-month mortality	8 (30)	2 (4)	0.003^*^	7.7	1.8–33.6
1-month mRS	5 (4–6)^**a**^	3 (2–4)^**a**^	0.0005^*^	–	–
3-month mRS	3 (2–6) ^**a**^	2 (1–3) ^**a**^	0.139	–	–

Abbreviations: *mRS* modified Rankin scale; *RR* elative risk; *CI* confidence interval

Unless specified, values are *p* values in non-parametric tests and values are numbers of patients (%)

^a^Median (interquartile range)

^*^Statistically significant

### Mortality of patients with persistent inflammation is 8-times higher than that of patients without persistent inflammation

Table 3 suggests that the incidence of pulmonary infection and 1-month mortality were 1.7 (95%CI 1.2–2.4) and 7.7 (95%CI 1.8–33.6) times higher in patients with persistent inflammation than in those without persistent inflammation.

### No relations between CRP and NLR at admission and CRP and 1-month outcomes

Given that CRP (high sensitivity CRP) is a widely applicable and representative indicator of systemic inflammation [18], we did additional studies on it and found no association between CRP and NLR at admission (see Supplementary Material), and grouping patients by CRP level according to the median (25–75% percentiles) = 1.2 (0.5–5) of our data and comparing short-term outcomes, we found no differences in pulmonary infection, urinary tract infection, 1-month mortality, and 1- and 3-month prognosis (Table 4).

**Table 4:** Comparisons between patients with high CRP and low CRP values

Short-term outcomes	CRP values		*p* value
	high	low	
Pulmonary infection	30 (47)	23 (46)	0.231
Urinary tract infection	15 (13)	6 (12)	0.950
1-month mortality	8 (30)	15 (29)	0.890
1-month mRS	4 (3–6)^**a**^	3 (2–4)^**a**^	0.431
3-month mRS	3 (2–6) ^**a**^	2 (1–3) ^**a**^	0.359

Abbreviations: *mRS* modified Rankin scale

Unless specified, values are *p* values in non-parametric tests and values are numbers of patients (%)

^a^Median (interquartile range)

^*^Statistically significant

## Discussion

Our single-centered prospective study suggested that persistent inflammation is associated with a poor prognosis for massive stroke, as well as high 1-month mortality and the occurrence of in-hospital pulmonary infections. In addition, we also demonstrated that higher baseline inflammation level predicts short-term poor functional outcome in massive stroke.

Persistent inflammation, as an informal but commonly used concept [14], can sometimes be understood as chronic inflammation, and to an extent adapted from non-resolving inflammation which is regarded as a significant and prolonged contributor to the pathology of diseases, such as atherosclerosis, obesity, cancer, chronic obstructive pulmonary disease, asthma, inflammatory bowel disease, neurodegenerative disease, multiple sclerosis, or rheumatoid arthritis [16]. Persistent inflammation has been extensively studied as a mechanism to explain chronic critical disease--a persistent but manageable organ dysfunction in the intensive care unit [15]; although there is no clear definition, similar studies have narrowed the concept because inflammation is not limited to organ failure but is present in any disease [19], infections or non-infections. The functional outcome of persistent inflammation in severe diseases has been poorly studied, leading to uncertainty about its precise definition and clinical significance.

Our study is the first of its kind to 1) apply circulatory NLR to assess the temporal and spatial persistence of inflammation and 2) continue our work on the inflammatory mechanisms of cerebral infarction, and this time in severe stroke investigate the significance and contribution of persistent inflammation. A similar attempt has been made to dynamically assess chronic kidney disease using C-reactive protein to get a time curve [20]. Our focus is the relationship to prognosis, not the repetition of the time curve of persistent inflammation. The advantage of NLR over a single marker such as C-reactive protein is that it is a combination of neutrophil and lymphocyte counts, both of which clearly play an important role in inflammatory pathways [16] and stroke pathology [3]. At the same time CRP has some disadvantages that perhaps lead to our negative results. 1) CRP is a protein not an inflammation cell and does not fully reflect the inflammatory process, it is only secreted by hepatocytes with an inflammatory state; 2) CRP is lagging in the inflammatory response and needs to be produced mainly by hepatocytes mediated by IL-6; 3) CRP is poorly measurable and in the face of inflammation CRP is generally elevated 10,000-fold, for example there is 0.8 mg/L elevated to 80,000 mg/L; 4) In fact, significant changes in CRP are only possible in the acute phase of severe infection or inflammation [18]. Persistent inflammation, a chronic exposure in severe stroke patients, will influence the evaluation, management and prognosis of the disease; we believe it will become a therapeutic target that can improve outcomes with only minimal means, such as controlling infection or controlling neutrophil or lymphocyte counts.

The underlying mechanism of NLR on stroke is due to a central role of neutrophils in all types of stroke. Circulating neutrophils are the first among various peripheral inflammatory cells to infiltrate the lesion (30 min to a few hours) [21] from the bone marrow and spleen, peak earlier (24–72 h) and decrease rapidly with time. This is associated with stroke severity [22], infarct size [23], and worse functional outcomes [24]. In the first 15 min of infiltration, neutrophils express endothelial adhesion molecules, P-selection glycoprotein ligand-1, lymphocyte function-associated antigen 1, and macrophage-1 antigen [25] and in 2 h they began to roll and adhere to the pial vessel of the brain; after 6–8 h, infiltration has begun [26], and peaked in 24–72 h [27]. Adhesion of neutrophils activate endothelium through adhesion molecules that promote neutrophil-endothelial interactions and neutrophil migration with the results of blood brain barrier disruption and brain edema [28]. In patients with stroke, the degree of neutrophil accumulation in cerebral ischemia lesions correlates with stroke severity and worse stroke outcome [29].

In contrast to neutrophils, lymphocytes decrease after ischemic stroke [30]. Lymphocytes including B and T cells, especially CD4+, CD8+ T cells and γδT cells play important roles in inflammation by producing pro-inflammatory cytokines e.g. interferon-γ and IL-17 [31], however Treg cell (CD4 + CD25 + Foxp3 + Treg cell) is a benefit for inflammation by releasing anti-inflammatory cytokines such as IL-10 that is neuroprotective via IL-10/JAK/STAT, PI3K, and MAPK pathways [29]. The decrease in the number of lymphocytes and increase in the number of neutrophils leads to increased NLR after stroke. Furthermore, an elevated NLR negatively impacts the functional outcome of patients with AIS due to secondary brain injury induced by neutrophil activation and due to increased risk of infection by lymphocyte suppression. After successful recanalization of large occluded vessels, the NLR falls with reperfusion of ischemic lesions. And it is reported that NLR decreased in 72 h post successful recanalization [32]. However, an elevated neutrophil count has noted as an independent predictor of poor outcome (mRS > 3) at 90 days despite TICI 2b/3 recanalization [32]. A study has found that higher NLR after thrombolysis is independently associated with symptomatic intracerebral hemorrhages and worse outcome at 3 months [11].

There are some limitations in the present study. Firstly, the present cohort study was conducted in a single stroke center and only recruited the Chinese population. Patients receiving thrombolysis were not randomly included, which might lead to selection bias. Secondly, massive stroke included severe ischemia and hemorrhage in brain in our study, however pathogenetic background of ischemic stroke and intracerebral hemorrhage is different and it can have influence on the results. The definition of persistent inflammation is our own criteria and is assessed using the two time point values of the NLR, which are relatively crude. There is a difference in the frequency and timing of blood collection in patients without thrombolysis and in patients with thrombolysis, which can have an impact on our sub-analysis. Thirdly, only NIHSS and mRS scores were used to evaluate functional outcomes of stroke patients. No other measures, such as advanced cognitive function, were used, which might compromise our conclusion.

## Conclusions

In our single-centered prospective study, we found that persistent inflammation is associated with a short-term poor functional outcome for massive stroke, as well as high 1-month mortality and the occurrence of in-hospital pulmonary infections. In addition, we found that higher baseline inflammation level predicts short-term poor functional outcome in massive stroke patients.

## Supplementary Information

**Additional file 1.**

## Data Availability

The datasets used and/or analyzed during the current study are available from the corresponding author on reasonable request.
