# Supplementary material for: Persistent inflammation worsens short-term outcomes in massive stroke patients
Source: BMC Neurol. 2021 Feb 10;21:62. doi: 10.1186/s12883-021-02097-9 (PMC7874622; doi:10.1186/s12883-021-02097-9)
Supplement: Supplementary file 1 — Additional file 1. [file 12883_2021_2097_MOESM1_ESM.pdf]

Supplemental material

Persistent Inflammation Worsens Short-term Outcomes in Massive Stroke Patients

Duanlu Hou[1], MS, Chunjie Wang[1,2], MS, Xiaofei Ye[3], PhD, Ping Zhong[4*], MD, Danhong Wu[1*], MD

1.Department of Neurology, The Fifth People's Hospital of Shanghai, Fudan University, Shanghai, China

2.Jiangchuan Community Health Service Center of Minhang District, Shanghai, China

3.Department of Health Statistics, Second Military Medical University, Shanghai, China

4.Department of Neurology, Shanghai Traditional Chinese Medicine Integrated Hospital Affiliated to Shanghai University of Chinese Medicine, Shanghai, China

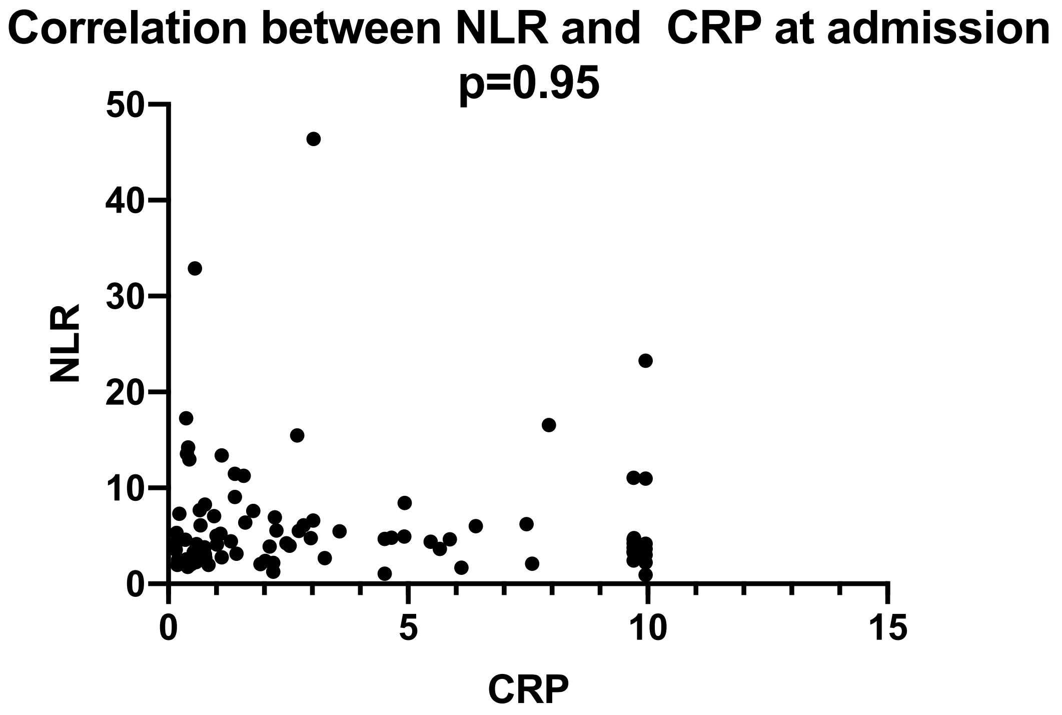
Correlation between NLR and CRP at admission
p=0.95
NLR
CRP
0
10
20
30
40
50
0
5
10
15
